# Complete mitochondrial genome sequence of *Leucoptera malifoliella* (Lepidoptera: Lyonetiidae)

**DOI:** 10.1080/23802359.2026.2616132

**Published:** 2026-01-21

**Authors:** Ruitao Yu, Shihang Zhao, Jiaqiang Zhao, Tianye Zhang, Lei Ma, Guoliang Xu, Nan Zhou

**Affiliations:** a Shijiazhuang Institute of Pomology, Hebei Academy of Agriculture and Forestry Sciences, Shijiazhuang, Hebei, P. R. China; b Lab of insect collection, Shijiazhuang Institute of Pomology, Hebei Academy of Agriculture and Forestry Sciences, Shijiazhuang, Hebei, P. R. China; c Chengde Agricultural and Rural Bureau, Chengde, Hebei, P. R. China; d Hebei Province Agricultural Products Quality and Safety Center, Shijiazhuang, Hebei, P. R. China

**Keywords:** Mitogenome, Yponomeutoidea, pear leaf blister moth, phylogeny

## Abstract

In this study, the complete mitochondrial genome of *Leucoptera malifoliella* (Costa, 1836) (Lepidoptera: Lyonetiidae) was sequenced and annotated. The genome is 15,214 bp in size, comprises 37 typical genes and a control region, and shows a positive AT skew. The gene content and order are similar to those of other lepidopterans. Phylogenetic analysis confirmed that families within Yponomeutoidea are monophyletic. The relationships among families are inferred as (Praydidae + Attevidae) + (Lyonetiidae + (Scythropiidae + Plutellidae)). This research improves our understanding of the relationships among major Yponomeutoidea lineages.

## Introduction

The pear leaf blister moth *Leucoptera malifoliella* (Costa, 1836) (Lepidoptera: Lyonetiidae), also known as *Leucoptera scitella* (Zeller, 1839) and *Cemiostoma scitella* (Zeller, 1848), is one of the most significant orchard pests in many temperate regions of Europe and Asia (Baufeld and Freier 1991; Mori and Wu 1991; Rovesti and Deseö 1991; Jenser et al. 1999; Seven 2006; Čirjak 2022). *L. malifoliella* prefers to feed on apples and pears but is polyphagous and damages leaves by forming circular mines (Béguinot 2017; Maccracken et al. 2021; Čirjak 2022). Heavy infestations can damage the leaves of host plants, resulting in premature leaf fall and reduced yield. Additionally, extensive tunneling of leaves may cause delays in shoot growth and decreased fruit weight. Repeated and heavy defoliation can ultimately weaken trees (Cravedi et al. 1992; Ovsyannikova and Grichanov, 2005; Nicolae et al. 2011). Considering the significance of this pest, the aim of this study was to sequence the complete mitogenome of *L. malifoliella*.

## Materials and methods

**Figure 1.**
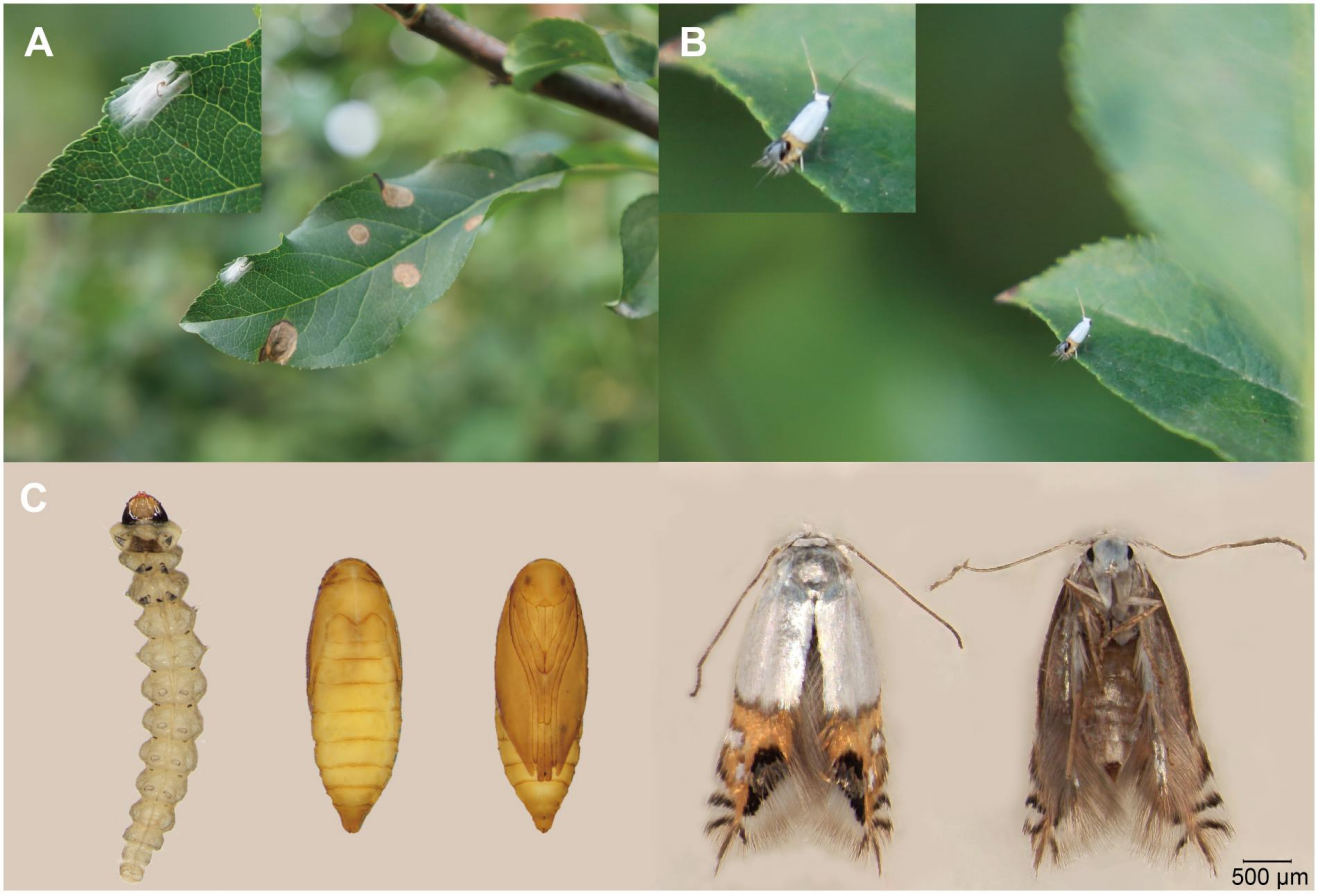
(A) Damaged apple leaves and a cocoon of *Leucoptera malifoliella* (inset). (B) Photograph of *L. malifoliella* in an apple orchard. (C) Larva, pupa, and adult stages of *L. malifoliella* (photographs taken by Ruitao Yu and Jiaqiang Zhao).

Adult specimens of *L. malifoliella* were collected from apple orchards surrounding the Shijiazhuang Institute of Pomology, Hebei Academy of Agriculture and Forestry Sciences, Shijiazhuang City, Hebei Province, China (38°12′N, 114°52′E) in July 2023 (Figure 1). The voucher specimens (voucher number: SGSLYO01; Url: http://www.hebnkysgs.com/; Contact person: Nan Zhou, znhbnky@163.com) were preserved in 95% ethanol, kept at −80 °C, and deposited in the insect collection of the Shijiazhuang Institute of Pomology.

The DNeasy Tissue kit (Qiagen) was used to extract the total DNA from specimens according to the manufacturer’s instructions. Furthermore, total genomic DNA was sequenced using the Illumina NovaSeq platform with paired-end reads of 2 × 150 bp at Personalbio Technology Co. Ltd. (Shanghai, China). The mitogenome was assembled and annotated using Geneious v9.0.2 (Kearse et al. 2012); the mitogenome of *Lyonetia clerkella* (NC037944) was used as the reference sequence. The protein-coding gene (PCG) boundaries were determined by identifying open reading frames through the invertebrate mitochondrial genetic code and alignment with reference sequences in Geneious v9.0.2. In addition, CGView Server (https://proksee.ca/) was used to generate mitogenome maps online (Grant et al. 2023). The sequencing depth and coverage map were drawn according to an online protocol (https://www.protocols.io/view/generating-sequencing-depth-and-coverage-map-for-o-4r3l27jkxg1y/v1) following Ni et al. (2023). The complete mitogenome sequence of *L. malifoliella* was described based on next-generation sequencing, with the aim of improving our understanding of the relationships among Yponomeutoidea families.

For phylogenetic analysis, PhyloSuite v1.2.3 software (Zhang et al. 2020; Xiang et al. 2023) was used to extract, align, and concatenate the genes. Phylogenetic relationships were estimated by best-fit partitioning schemes and substitution models, using both Bayesian inference and maximum likelihood methods based on the PCG123 dataset (all codon positions of the 13 PCGs) in PhyloSuite v1.2.3.

## Results

**Figure 2.**
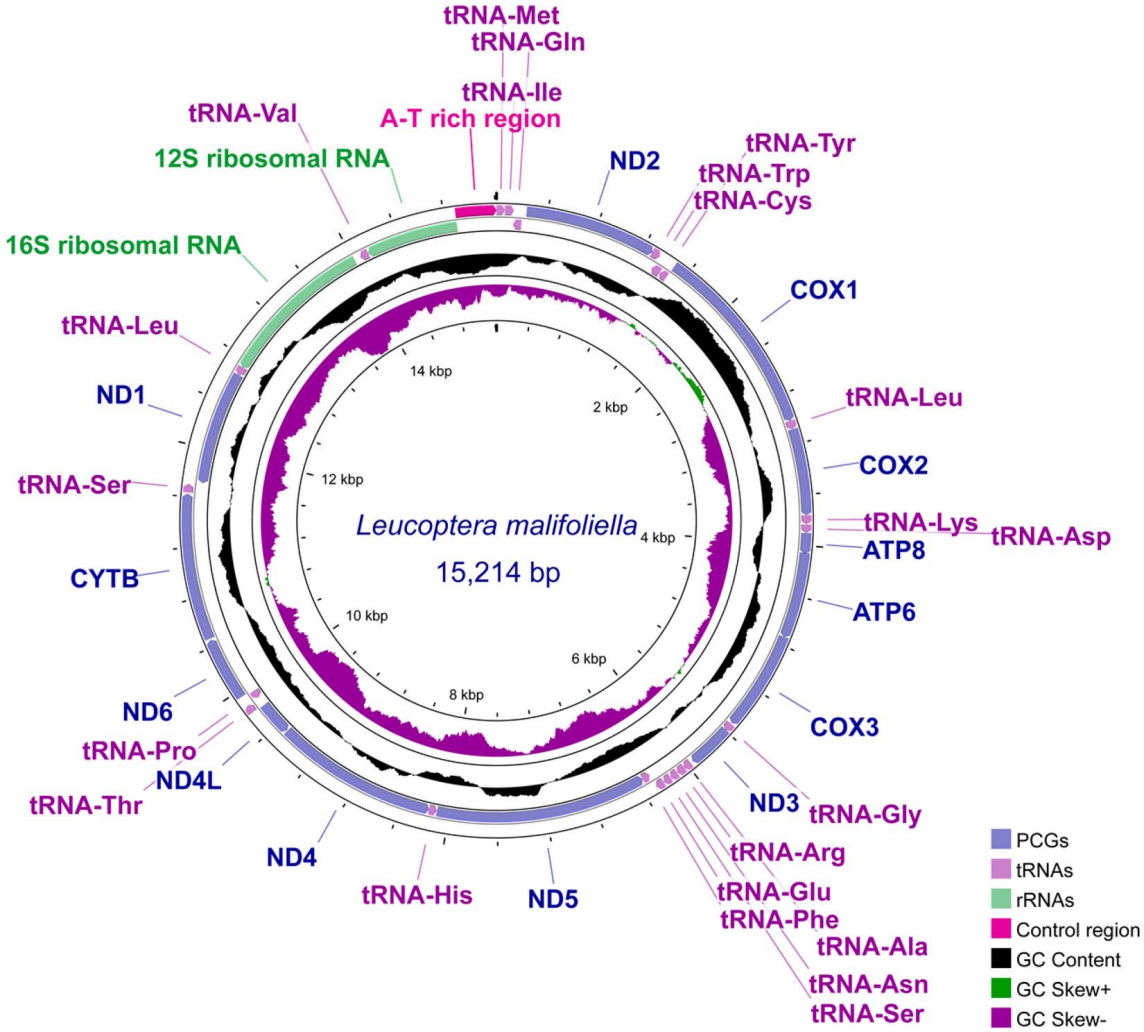
Gene map of the mitochondrial genome of *L. malifoliella.* Genes encoded on heavy and light strands with inverse arrow directions are shown outside and inside the circle, respectively. Genes are color coded in the outer circles. GC content is indicated in black in the inner circle.

The annotated genomic sequence (submitted to GenBank under accession number OR438296) is a closed circular molecule, 15,214 bp in length, with an average sequencing depth of 383.17x (Figure S1), comprising 37 typical genes (Figure 2). Of the 37 genes, 23 are encoded on the heavy (+) strand and the remainder are encoded on the light (-) strand. Of the 13 PCGs, 12 contain the typical start codon ATN (five ATA, four ATG, and three ATT), whereas *cox1* is initiated by codon CGA. Nine PCGs have the most common stop codon, TAA, two PCGs have stop codon TAG (*nad4L* and *nad6*), and two PCGs have an incomplete termination codon T (*cox1* and *cox2*). All 22 transfer RNAs can fold into the characteristic cloverleaf secondary structure; however, *trnS1* lacks a dihydrouridine arm, as identified in other Yponomeutoid species. Moreover, the mitogenome of *L. malifoliella* is biased toward a high representation of the nucleotides A (41.8%) and T (40.5%) and a low representation of the nucleotides C (10.6%) and G (7.1%), showing a positive AT skew and a negative GC skew.

**Figure 3.**
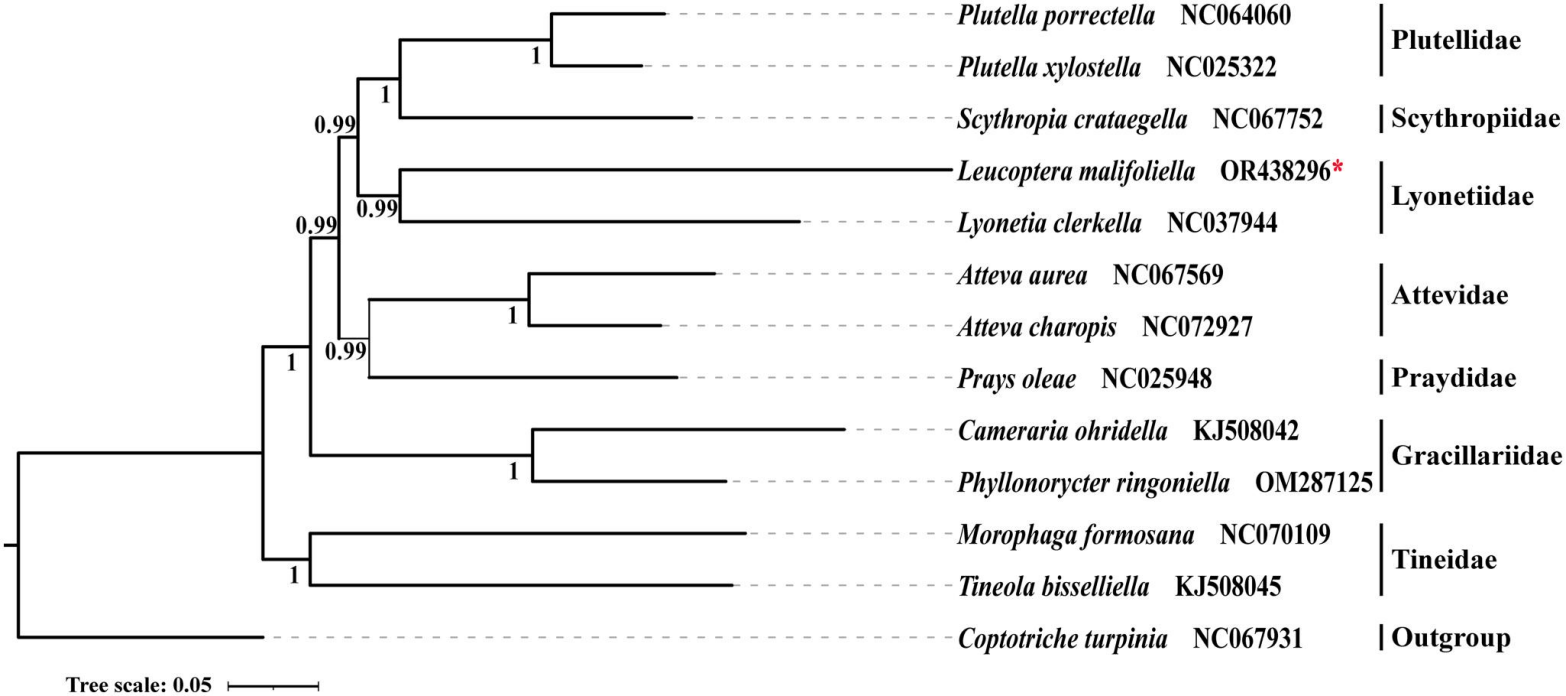
Phylogenetic relationship of *L. malifoliella* inferred by Bayesian inference based on the concatenated dataset of 13 PCGs. Numbers on nodes denote the Bayesian posterior probabilities. Maximum likelihood analyses showed the same topology (data not shown). The following sequences were used: *Plutella porrectella* NC064060 (unpublished), *Plutella xylostella* NC025322 (Dai et al. 2016), *Scythropia crataegella* NC067752 (unpublished), *Leucoptera malifoliella* OR438296 (this study), *Lyonetia clerkella* NC037944 (unpublished), *Atteva aurea* NC067569 (unpublished), *Atteva charopis* NC072927 (unpublished), *Prays oleae* NC025948 (van Asch et al. 2016), *Cameraria ohridella* KJ508042 (Timmermans et al. 2014), *Phyllonorycter ringoniella* OM287125 (Zhou et al. 2022), *Morophaga formosana* NC070109 (unpublished), *Tineola bisselliella* KJ508045 (Timmermans et al. 2014), *Coptotriche turpinia* NC067931 (unpublished).

Bayesian inference and maximum likelihood analyses generated similar topologies and showed that most major groups are consistently monophyletic (Figure 3). Phylogenetic analyses indicated that Yponomeutoidea is monophyletic with respect to Gracillarioidea and revealed a close relationship between Praydidae and Attevidae. Families within the Yponomeutoidea were recovered as monophyletic groups. The relationships among families were inferred as (Praydidae + Attevidae) + (Lyonetiidae + (Scythropiidae + Plutellidae)).

## Discussion and conclusion

The complete mitochondrial genome of *L. malifoliella* has a quadripartite structure of 15,214 bp, containing 13 PCGs, 22 transfer RNA genes, two ribosomal RNA genes, and one control region. The mitogenome is similar to that of other species in the Yponomeutoidea superfamily. The results of this study confirm that Yponomeutoidea is monophyletic with respect to Gracillarioidea, and that a close relationship exists between Praydidae and Attevidae, which is consistent with previous research (Ulenberg 2009; Sohn et al. 2013; Lewis and Sohn 2015). However, the phylogenetic position of Scythropiidae remains controversial (Sohn et al. 2013). Because of the limited availability of complete mitochondrial genomes, further investigation is required to validate the monophyly of families within Yponomeutoidea. Further mitogenome sequencing is expected to yield increasingly robust estimates of relationships among the major lineages of Yponomeutoidea.

## Supplementary Material

Supplementary Figure 1.png

## Data Availability

The data that support the findings of this study are openly available in GenBank of NCBI at https://www.ncbi.nlm.nih.gov, reference number OR438296. The associated BioProject accession number, SRA data, and BioSample accession number are PRJNA1004856, SRR25620967 and SAMN36957541, respectively.
