# Supplementary figures and images for: Complete mitochondrial genome sequence of Leucoptera malifoliella (Lepidoptera: Lyonetiidae)
Source: Mitochondrial DNA B Resour. 2026 Jan 21;11(2):272–5. doi: 10.1080/23802359.2026.2616132 (PMC12825583; doi:10.1080/23802359.2026.2616132)

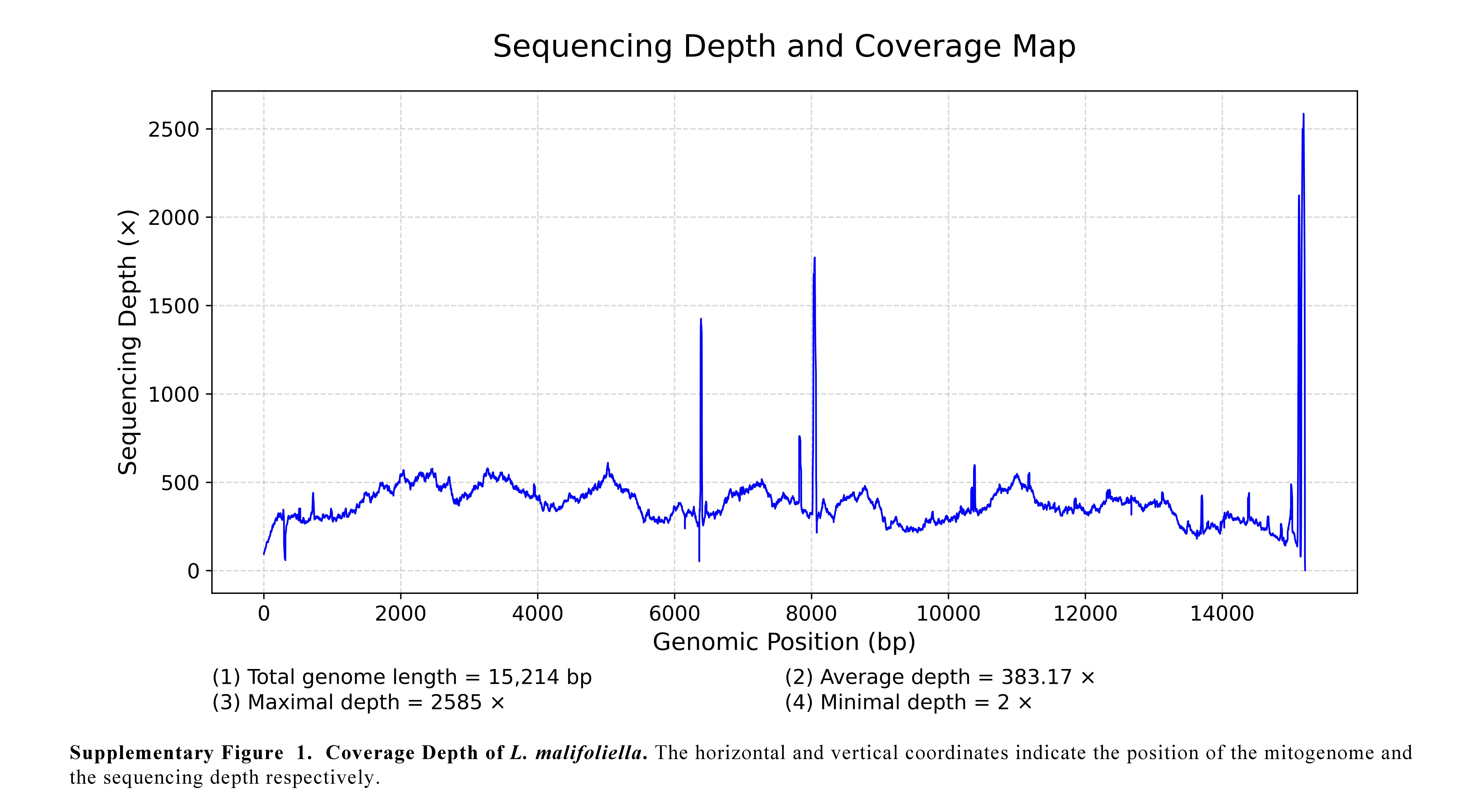

Supplement: Supplementary Figure 1.png [file TMDN_A_2616132_SM9364.png]
